# Effect of Growth Hormone Treatment on the Concentration of Selected Metabolic Markers in Girls With Turner Syndrome

**DOI:** 10.3389/fendo.2022.818735

**Published:** 2022-06-13

**Authors:** Ewa Błaszczyk, Jakub Gawlik, Joanna Gieburowska, Agnieszka Tokarska, Małgorzata Kimsa-Furdzik, Grzegorz Hibner, Tomasz Francuz, Aneta Gawlik

**Affiliations:** ^1^ Department of Pediatrics and Pediatric Endocrinology, Faculty of Medical Sciences in Katowice, Medical University of Silesia, Katowice, Poland; ^2^ Student Scientific Society, Department of Biophysics, Jagiellonian University Medical College, Kraków, Poland; ^3^ Department of Biochemistry, Faculty of Medical Sciences in Katowice, Medical University of Silesia, Katowice, Poland

**Keywords:** Turner syndrome, MMP-1 (matrix metalloproteinase-1), MMP-2 (matrix metalloproteinase-2), MMP-9 (matrix metalloproteinase-9), VEGF (vascular endothelial growth factor), GDNF (glial cell line-derived neurotrophic factor), BDNF (brain-derived neurotrophic factor)

## Abstract

**Background:**

As Turner syndrome (TS) predisposes to obesity and metabolic disorders, and their complications, such as cardiovascular diseases, are the main causes of shortened life expectancy in patients with TS, new metabolic markers that could serve as early predictors of dysmetabolic state are sought.

**Objective:**

Assessment of MMP-1 (matrix metalloproteinase-1), MMP-2 (matrix metalloproteinase-2), MMP-9 (matrix metallopeptidase-9), BDNF (brain-derived neurotrophic factor), GDNF (glial cell line-derived neurotrophic factor), and VEGF (vascular endothelial growth factor) before the onset of growth hormone (GH) therapy and then during GH treatment as well as markers assessment during GH medication in girls with TS to establish marker stability and repeatability, and the impact of GH on markers concentration.

**Method:**

The concentrations of circulating MMP-1, MMP-2, MMP-9, BDNF, GDNF, and VEGF were measured in nine girls with TS before the onset of GH therapy and then after at least 3 months of treatment period. Subsequently, markers concentration was determined in 17 girls during GH medication, with the first determination after at least a 3-month treatment period. The patients’ clinical and biochemical phenotypes were determined by weight, height, BMI, total cholesterol, HDL cholesterol, triglycerides, and glucose concentration.

**Results:**

Comparison of markers concentration revealed a significantly higher concentration of MMP-2 in patients undergoing GH treatment (132.1 ± 42.05) than before the onset of therapy (105.0 ± 45.5, p=0.045). The values of the first measurement of VEGF in girls with TS undergoing GH therapy were significantly higher than those during the second measurement (30.9 ± 33.4 vs. 12.5 ± 11.7, p=0.029). There were no statistically significant differences between the measurements of the remaining markers concentration at any stage of the analysis.

**Conclusion:**

Increase in MMP-2 concentration is visible during GH therapy in comparison to the pre-GH period in girls with TS which demands confirmation in subsequent tests. The role of VEGF requires further studies in the context of carbohydrate-lipid disturbances in girls with TS and its association with GH treatment.

## Introduction

Obesity is a major risk factor for many other metabolic disorders, and can also lead to the development of cardiovascular diseases and, consequently, to increased mortality (1). Metabolic syndrome (MetS) components occur more frequently in Turner syndrome (TS), taking into account the period of childhood (2). Moreover, heart defects are more common in TS than in the general population (3). All these factors lead to a shorter life expectancy in patients with TS (4). It is still uncertain whether higher cardiometabolic risks in TS are more a consequence of intrinsic factors or the result of modifiable metabolic risk factors connected with lifestyle. Therefore, the search for new markers as potential early predictors of the natural development of a dysmetabolic state seems to be reasonable. A better understanding of what affects markers expressions can help find more detailed targets of potential actions in the future.

Therefore, our work focuses on the assessment of concentrations of brain-derived neurotrophic factor (BDNF), glial cell line-derived neurotrophic factors (GDNF), vascular endothelial growth factor (VEGF), and matrix metalloproteinases (MMPs) as potential early markers of dysmetabolic state. Our previous study was performed on 17 patients with TS undergoing growth hormone (GH) therapy (5). Subsequently, we re-examined the markers concentration before the onset of GH therapy to exclude its potential influence (6).

BDNF, a member of the nerve growth factor family, is also involved in weight regulation and energy expenditure by reducing appetite (7). The concentration of BDNF correlates negatively with body weight (8) and age (9); therefore, it is believed to be an anti-aging factor (6). BDNF affects glucose and lipid metabolism and is also considered an anorexigenic factor (10). Moreover, significantly lower plasma BDNF concentrations were observed in patients with MetS. It also appears that physical activity may alter the concentration of BDNF (11). These findings support the metabotropic deficit hypothesis (12, 13). Most likely, some BDNF genotypes resulting from BDNF gene polymorphism can be recognized as a potential factor in the development of obesity and insulin resistance (14, 15). Our previous studies revealed higher BDNF levels in girls with TS, both before the onset of GH therapy (5) as well as during treatment (6) compared to the control group. Interestingly, the assessment of BDNF concentration in adult TS patients by other authors confirms its higher concentration in patients with this syndrome (16).

GDNF is responsible for survival of neurons, protecting them from damage (17), and plays a neuroprotective role for catecholaminergic and sympathetic neurons, taking part in the regulation of intake and energy expenditure (18). Transgenic mice with overexpression of GDNF in glial cells are protected from obesity, glucose intolerance, insulin resistance, and hepatic steatosis caused by high-fat meals (19). Administration of GDNF can result in weight loss in humans (20). Mentioned reports suggest GDNF has a protective role against the development of major components of the metabolic syndrome. Like BDNF, GDNF signs in the suggested metabotropic hypothesis of metabolic syndrome.

VEGF is involved in embryogenesis, wound healing, tumor metastasis, rheumatoid arthritis, and formation of new blood vessels from pre-existing vessels (21), and appears to have a metabolic effect. Its concentration is lower in obese people, whose blood vessel development is insufficient. This results in hypoxia of adipocytes and local inflammation (22). Hypoxia leads to glucose intolerance (23), while ongoing low-grade inflammation causes insulin insensitivity (24), which is associated with metabolic syndrome. Transgenic mice with VEGF overexpression are protected against the development of obesity, as well as glucose intolerance and insulin resistance induced by diet, resulting from local hypoxia (25). However, data regarding VEGF levels and metabolic processes are inconclusive. One study revealed a positive correlation between the concentrations of circulating VEGF levels and BMI (26), and another study reported increased levels of VEGF in overweight and obese people (27), which contradicts the above-mentioned reports.

MMPs are a group of enzymes that degrade extracellular matrix components with activity influenced by growth factors, ongoing inflammation, or inhibitors of metalloproteinases (28). MMPs play an important role in angiogenesis and wound healing (29) and participate in the modulation of adipogenesis (30). MMP-1 seems to be involved in a process facilitating the development of adipose tissue, leading to obesity (31). Plasma concentrations of MMP-2 and MMP-9 are increased in patients with metabolic syndrome (32, 33). Moreover, MMP-2 and MMP-9 levels were elevated in patients with isolated systolic hypertension as compared to the control group (34, 35). In our previous study, MMP-1 was significantly higher in the study group, with a significant positive correlation with Z-score BMI (5), while studies before GH treatment revealed lower concentrations of MMP-2 in girls with TS compared to healthy girls with short stature (6).

Here, in the context of higher cardiometabolic risk in TS, we aimed to analyse the influence of GH therapy on discussed above markers and to monitor their stability and repeatability during GH therapy.

## Patients and Methods

**Figure 1:**
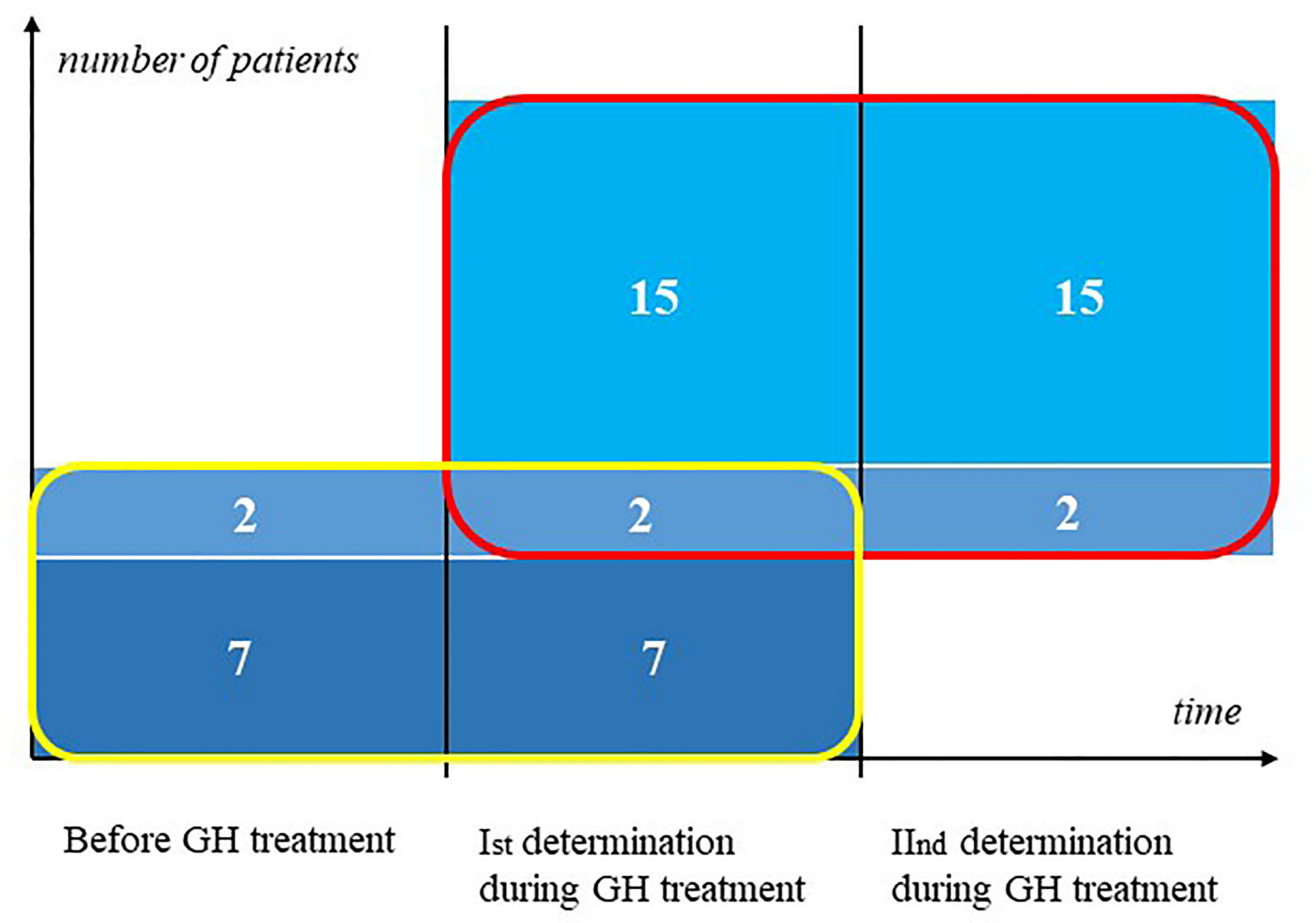
Groups of Patient participating in the study.

First, we determined markers concentration in nine girls with TS before the onset of GH therapy and then after at least a 3-month treatment period, before the end of the first year of therapy. Subsequently, we evaluated markers concentration in 17 girls during GH medication, the first measurement was performed after at least 3 months of treatment. The second measurement was performed after at least 3 months from the previous measurement (2 patients were assessed between 6 months to 1 year from the previous visit, 10 patients – between 1 to 2 years, and 5 patients had second determination after 2 years from first determination). 2 patients are included in both groups, they have a triple determination of markers. The other patients do not overlap (**Figure 1**). The diagnosis of Turner syndrome was confirmed by karyotyping with routine G-banding according to the recommendations of the American College of Medical Genetics.

### Clinical Phenotype of Study Participants

The detailed anthropometric analysis was based on weight and height measurements, along with body mass index (BMI) calculation, using the standard formula of weight (kg) divided by height (m) squared. Weight was measured with a Seca scale with a precision of 100 g and height with a Harpenden stadiometer with a graduation of 0.1 cm. A BMI above the 97th percentile was classified as obese, while a BMI between the 90th and 97th percentiles as overweight based on the BMI chart for healthy girls (36). Based on the age, sex, BMI, and appropriate reference standard, the BMI Z-score was calculated using the international (International Obesity Task Force; IOTF) body mass index (BMI) cut-offs (37). The Tanner scale was used to assess puberty (38). Arterial pressure was determined in measurements with a sphygmomanometer or 24-hour monitoring of arterial pressure was performed and hypertension was diagnosed according to Blood Pressure Values Park’s Pediatric Cardiology for Practitioners (https://doctorlib.info/cardiology/).

### Biochemical Phenotype of Study Participants

Morning fasting venous blood samples were collected to measure MMP-1, MMP-2, MMP-9, BDNF, GDNF, and VEGF. The concentrations of these markers were determined by sandwich ELISA using kits distributed by R&D systems.

Concentrations of total cholesterol (TCh), HDL cholesterol (HDL-chol), and triglycerides (TG) were analyzed enzymatically (Beckman Coulter, Brea, CA). An enzymatic test (hexokinase method) was used for the quantitative determination of glucose (Beckman Coulter). Insulin levels were determined using a chemiluminescence immunoassay using an IMMULITE 2000 analyzer. An oral glucose load test of 1.75 g/kg was performed (dose of 75 g was maximal), with the determination of glucose and insulin levels at two time points: 0’ and 120’. Fasting insulin-to-glucose ratio indices of the IR - the quotient of insulin concentration to fasting glucose >0.3 was considered as insulin resistance (39).

To assess the concentration of TG, HDL-chol, and fasting glucose in children aged 3–11 years, the results were categorized according to the IDEFICS study (40): HDL-chol <= 10^th^ percentile or triglycerydes >= 90^th^ percentile, fasting glucose >= 90^th^ percentile. Hypertension was evaluated according to Blood Pressure Values Park’s Pediatric Cardiology for Practitioners and obesity was recognized while BMI was above 97 centile according to BMI charts for healthy girls (36).

For older children, IDF cut-off points were used (41): triglycerides >= 150 mg/dL, HDL- chol <40 mg/dl, glucose >=100 mg/dl; for recognition of hypertension and obesity we applied criteria as mentioned above.

MetS was recognized if a critical value was exceeded for three or more of these risk factor.

For technical reasons 2 out of 9 patients had no data on lipids and glucose before GH treatment.

### Statistical Analysis

Data processing and statistical analyses were performed using Statistica 13 PL software. Statistical significance was set at P < 0.05. Normality was assessed using the Kolmogorov–Smirnov test. Variables with a normal distribution were analyzed using t-tests and reported as mean ± standard deviation (SD); those without normal distribution were analyzed using the Mann–Whitney U test and reported as median with interquartile range (IQR).

The study was conducted in accordance with the Declaration of Helsinki and approved by the Ethics Committee of the Medical University of Silesia (resolution number KNW/0022/KB1/162/15/16)). Informed consent was obtained from each participant aged over 16 years, a parent, or a legal guardian.

## Results

### Comparison Before and During GH Treatment

Comparison of the analyzed markers concentration revealed a significantly higher concentration of MMP-2 in patients undergoing GH treatment than before the onset of therapy (132.1 ± 42.05 vs. 105.0 ± 45.5 ng/ml, p=0.045). In almost all patients, the measurement during treatment showed a higher concentration of MMP-2. Two patients who had the highest MMP-2 values before starting treatment also had the highest levels during GH therapy. One of the girls had hypertension (**Table 1** – Patients No. 4 and 5).

**Table 1:** Detailed results of metabolic parameters before GH treatment.

Patient no.		1	2	3	4	5	6	7	8	9
Categorical variables										
BMI [percentile]	V1	10 - 25	25 - 50	75 - 90	10 - 25	25 - 50	90 - 97	> 97	10 - 25	10 - 25
	V2	25	25-50	75 -90	10 - 25	50	90 – 97	> 97	25 - 50	10 - 25
HD [0/type]		0	BAV	0	0	CoA	0	0	0	0
HT [0/1]	V1	0	0	1	0	1	0	0	0	0
	V2	0	0	1	0	1	0	0	0	0
hyperTG [0/1]	V1	–	0	0	0	–	0	0	0	0
	V2	0	0	1	0	0	0	1	0	0
LowHDL [0/1]	V1	–	0	0	0	–	0	0	0	0
	V2	0	0	0	0	0	0	0	0	0
IFG [0/1]	V1	1	0	0	0	–	0	0	–	1
	V2	0	1	1	0	0	0	1	0	1
**Continuous variables**										
BMI Z-score	V1	-1.45	-0.39	1.09	-0.87	0.05	1.83	2.38	-0.81	-0.89
	V2	-0.7	-0.11	1.15	-0.75	0.25	1.59	2.36	-0.68	-0.72
BDNF [pg/ml]	V1	36766.14	35399.83	29643.89	25890.17	21624.09	30259.18	62615.94	24109.61	47195.14
	V2	5389.13	35330.78	34411.43	26217.21	30835.77	35230.69	59340.27	28231.39	56472.23
VEGF [pg/ml]	V1	24.65	3.68	2.31	6.51	15.72	5.87	21.05	15.62	16.27
	V2	114.96	2.89	2.11	8.18	8.86	6.42	10.04	16.25	18.6
MMP-1 [pg/ml]	V1	1212.28	1096.54	5381.46	1390.7	3590.28	1891.16	2265.13	1804.02	2386.6
	V2	198.33	877.01	5724.48	1346.91	3498.86	1890.72	2484.82	1185.24	2432.92
MMP-2 [ng/ml]	V1	65.28	57.84	102.37	189.3	164.58	75.66	74.29	95.77	119.69
	V2	167.85	88.06	125.32	209.68	173.05	96.88	128.43	98.09	104.27
MMP-9 [ng/ml]	V1	69.6	222.4	38.69	108.41	95.04	99.69	619.06	100.36	255
	V2	63.73	283.41	59.95	180.77	130.02	145.77	363.3	180.98	152.22

HD, congenital heart disease; BAV, bicuspid aortic valve; CoA, coarctation of the aorta; HT, hypertension; hyperTG, hypertriglicerydemia; low HDL, low HDL cholesterol concentration; IFG, impaired fasting glucose; MMP-1, matrix metalloproteinase-1; MMP-2, matrix metalloproteinase-2; MMP-9, matrix metallopeptidase 9; BDNF, brain-derived neurotrophic factor; VEGF, vascular endothelial growth factor; (-), missing result; V1, first determination (before GH treatment); V2, second determination (during GH treatment); Parameters in the [0/1 system], [meets/does not meet the recognition criteria].

There were no statistically significant differences in the concentrations of the remaining markers before and during GH treatment. Detailed data are presented in **Table 2**.

**Table 2:** Clinical and biochemical parameters of patients before and during GH treatment.

	Group of patients before GH treatment n=9	Group of patients during GH treatment n=9	P value
Age [years]	9.03 ± 3.57	9.40 ± 3.41	<0.001
Weight [kg]	28.1 ± 14.61	29.73 ± 14.72	0.008
Height [cm]	120.43 ± 19.79	123.59 ± 20.33	0.003
BMI [kg/m2]	15.60 (14.72 – 22.27)	16.02 (15.04 – 22.07)	0.045
BMI Z-Score	-0.01 ± 1.52	-0.11 (-0.72 – 1.15)	NS
hSDSTS	1.10 ± 1.12	1.52 ± 1.15	0.037
Glucose 0’ [mg/dl]	89.86 ± 7.43	92.13 ± 10.37	NS
Glucose 120’ [mg/dl]	112.00 ± 21.28	124.63 ± 24.23	NS
Insulin 0’ [IU/l]	4.90 (2.00 – 7.50)	14.04 ± 9.08	NS
Insulin 120’ [IU/l]	45.41 ± 40.96	46.65 (35.55 – 107.00)	0.023
Insulin/glucose ratio	0.06 ± 0.03	0.15 ± 0.10	NS
TCh [mg/dl]	187.71 ± 23.76	179.50 ± 30.81	NS
HDL [mg/dl]	52.67 ± 7.18	55.45 ± 10.19	NS
LDL [mg/dl]	121.00 ± 20.13	110.05 (80.75 – 125.90)	NS
TG [mg/dl]	70.00 ± 30.40	69.00 (61.00 – 102.00)	0.023
TSH [IU/ml]	2.71 (2.70 – 3.06)	2.78 (2.70 – 3.76)	NS
fT4 [ng/dl]	1.50 ± 0.25	1.34 ± 0.31	NS
IGF-1 [ng/ml]	177.10 ± 89.01	307.59 ± 199.40	NS
CRP [mg/l]	1.15 (0.60 – 2.10)	0.20 (0.20 – 0.30)	NS
ALT [IU/l]	18.38 ± 3.11	19.00 ± 5.94	NS
AST [IU/l]	30.75 ± 8.15	34.13 ± 7.22	NS
VEGF [pg/ml]	15.62 (5.87–16.27) *[2.31 – 24.65]	8.86 (6.42 – 16.25) *[2.11 – 114.96]	NS
MMP-9 [ng/ml]	100.36 (95.04 – 222.40) *[38.69 – 619.06]	152.22 (130.02 – 180.98) *[59.95 – 363.30]	NS
BDNF [pg/ml]	30259.18 (25890.17 – 36766.14) *[21624.09 – 62615.94]	34411.43 (28231.39 – 35330.78) *[5389.13 – 59340.27]	NS
MMP-1 [pg/ml]	1891.16 (1390.70 – 2386.60) *[1096.54 – 5381.46]	2182.143 ± 1652.712 *[198.33 – 5724.48]	NS
MMP-2 [ng/ml]	105.0 ± 45.50 *[57.84 – 189.30]	132.40 ± 42.05 *[88.06 – 209.68]	0.045

GDNF concentration was below detection limit. Data are presented as mean ± SD (T-test) or median with interquartile range (Mann–Whitney U test) and for metabolic markers (VEGF-MMP-2) data are also presented as minimum – maximum ranges [*]. hSDSTS, hSDS according to growth chart for Turner syndrome; BMI Z-score, body mass index Z-score; TCh, total cholesterol, HDL, HDL cholesterol, LDL, LDL cholesterol; TG, triglycerides; TSH, thyroid-stimulating hormone; fT4, free T4; IGF-1, insulin-like growth factor 1; CRP, C-reactive protein; ALT, alanine transaminase; AST, aspartate transaminase; MMP-1, matrix metalloproteinase-1; MMP-2, matrix metalloproteinase-2; MMP-9, matrix metallopeptidase 9; BDNF, brain-derived neurotrophic factor; VEGF, vascular endothelial growth factor; NS, not significant.

It was noted that two clearly highest concentrations of MMP-1 before GH (5381.46 and 3590.28 pg/ml) belonged to two patients with elevated blood pressure values. They had the highest levels of MMP-1 during GH therapy as well (5724.48 and 3498.86 pg/ml). We observed development of metabolic disorders in one of these patients during GH treatment (**Table 1** – Patient no.3).

In turn, the concentration of MMP-9 was clearly higher in one patient, both before GH and during therapy, compared to the rest. She was the only obese girl who qualified for the study, who additionally had impaired fasting blood glucose (IFG) on the second test (**Table 1**, Patient no.7).

Furthermore, remarkably higher BDNF concentrations were obtained in two patients. These were the same two patients with the highest values, both before and during the inclusion of GH. For the highest pre-GH result, it belonged to the only obese patient enrolled in the study (**Table 1**, Patient no.7), and the second belonged to girl with IFG (**Table 1**, Patients no.9). During treatment, the highest result was found in the obese patient who additionally revealed IFG (**Table 1**, Patient no.7), and the second to the same patient as the previously recognized IFG (**Table 1**, Patients no.9).

For VEGF concentrations, there were no potential associations with the presence of components of metabolic syndrome.

GDNF concentrations were below the limit of detection in all patients.

The clinical and biochemical characteristics of the participants presenting the frequency of the components of metabolic syndrome are presented in **Table 3**, all available clinical and biochemical parameters of patients are included in **Table 2**.

**Table 3:** Components of MetS in TS patients before and during GH treatment (2 out of 9 patients had no data on lipids and glucose before GH treatment).

Component of MetS	Group of patients before GH treatment (n=9)	Group of patients during GH treatment (n=9)
obesity	1	1
hypertension	2	2
IFG	2	4
low HDL	0	0
hypertriglyceridemia	0	2

MetS, metabolic syndrome; IFG, impaired fasting glucose.

Among the biochemical parameters, statistically significant differences were found between first and second determination of the concentration of triglycerides and insulin determined at 120 minute of the glucose load test. No statistically significant differences between the determinations were found between the other parameters. Detailed data is presented in **Table 2**. All patients had a fasting insulin/glucose ratio of <0.3. The concentration of TCh did not correlate with any of the metabolic markers in any of the analyzed groups.

According to the Tanner scale, two patients before the onset of GH therapy started puberty spontaneously (karyotype: 1^st^ patient - 45,X[17]/46,XX [33] and 2^nd^ patient – 45,X). The 3^rd^ one with 45,X started induced puberty at the time of the second markers determination:Tanner stage = B2, no more advanced puberty characteristics. The onset of puberty was not recorded in any of the remaining participants. Heart defects were observed in two patients: coarctation of the aorta (1 patient) and bicuspid aortic valve (1 patient).

### Comparison During GH Treatment

The only statistically significant difference was observed between the first and second VEGF measurements. The values of the first measurement were statistically significantly higher (30.9 ± 33.4 vs. 12.5 ± 11.7 pg/ml, p=0.029). There were no statistically significant differences between the measurements of the remaining markers concentration (**Table 4**). In the first evaluation (2 patients) and in the second evaluation (3 patients) had VEGF levels were below the detection limit.

**Table 4:** Clinical and biochemical parameters of patients before and during GH treatment.

	First measurement measurement during GH treatment *n=17*	Second measurement measurement during GH treatment *n=17*	P value
Age [years]	8.36 ± 3.16	10.77 ± 3.10	<0.001
Weight [kg]	25.02 ± 9.21	33.35 ± 8.36	NS
Height [cm]	117.82 ± 16.55	132.86 ± 12.20	<0.001
BMI [kg/m2]	16.72 (15.47 – 18.56)	18.59 ± 2.24	NS
BMI Z-Score	0.37 ± 0.99	0.52 ± 0.82	NS
hSDSTS	1.22 ± 1.11	2.11 ± 1.02	<0.001
Glucose 0’ [mg/dl]	90.00 (85.00 – 95.00)	90.24 ± 7.61	NS
Glucose 120’ [mg/dl]	103.00 (98.00 – 123.00)	119.31 ± 23.77	NS
Insulin 0’ [IU/l]	8.24 ± 5.02	11.30 ± 4.64	NS
Insulin 120’ [IU/l]	38.37 ± 21.57	53.80 (48.20 – 80.00)	NS
Insulin/glucose ratio	0.09 ± 0.05	0.12 ± 0.05	NS
TCh [mg/dl]	168.29 ± 35.27	172.00 (156.00 – 190.00)	NS
HDL [mg/dl]	61.35 ± 9.82	63.44 ± 10.29	NS
LDL [mg/dl]	96.61 ± 23.53	79.45 (76.15 – 102.80)	NS
TG [mg/dl]	69.00 (57.00 – 91.00)	74.00 ± (58.50 – 100.00)	NS
TSH [IU/ml]	3.29 (2.72 – 4.02)	2.47 (2.22 – 3.88)	NS
fT4 [ng/dl]	1.42 (1.27 – 1.49)	1.33 ± 0.19	NS
IGF-1 [ng/ml]	397.38 ± 199.34	501.38 ± 256.03	<0.001
CRP [mg/l]	0.30 (0.20 – 0.40)	0.40 (0.30 – 1.10)	NS
ALT [IU/l]	16.00 (15.00 – 20.50)	21.35 ± 7.11	NS
AST [IU/l]	30.0 (24.50 – 33.00)	30.94 ± 7.25	NS
VEGF [pg/ml]	30.94 ± 33.43	12.46 ± 11.69	0.029
MMP-9 [ng/ml]	260.24 ± 150.2279	264.39 ± 186.2421	NS
BDNF [pg/ml]	31271.20 ± 12176.04	30568.38 ± 9005.71	NS
MMP-1 [pg/ml]	2085.39 (1229.22 – 5250.84)	2007.4 (1482.52 – 2847.36)	NS
MMP-2 [ng/ml]	100.73 (86.06 – 122.4)	99.8 (85.44 – 116.8)	NS

GDNF concentration was below detection limit. Data are presented as mean ± SD (T-test) or median with interquartile range (Mann–Whitney U test). hSDSTS, hSDS according to growth chart for Turner syndrome; BMI Z-score, body mass index Z-score; TCh, total cholesterol, HDL, HDL cholesterol, LDL, LDL cholesterol TG, triglycerides;TSH, thyroid-stimulating hormone; fT4, free T4; IGF-1, insulin-like growth factor 1; CRP - C-reactive protein; ALT, alanine transaminase; AST, aspartate transaminase; MMP-1, matrix metalloproteinase-1; MMP-2, matrix metalloproteinase-2; MMP-9, matrix metallopeptidase 9; BDNF, brain-derived neurotrophic factor; VEGF, vascular endothelial growth factor; NS, not significant.

In the case of MMP-1, we noted several clearly higher (above 6000.00 pg/ml), repeatable marker concentrations. Girl with highest level in the first and second determination had coexisting hypertension and during second determination she had elevated insulin level in 120 minute of oral glucose load test, however second highest level in the first and second measurement belonged to the girl without any metabolic syndrome components. The third highest measurement in first determination was found in girl with hypertriglicerydemia, however level of MMP-1 was not so high in second determination.

For the remaining markers, it was not noticed that the extreme values were related to the more frequent occurrence of metabolic disorders.

GDNF concentrations were below the limit of detection in all patients.

The clinical and biochemical characteristics of the participants presenting the frequency of the components of metabolic syndrome are presented in **Table 5**. All available clinical and biochemical parameters of patients are included in **Table 4**. Statistically significant differences were found in IGF-1 concentrations between the measurements, with an increase during the second measurement (p<0.001). All patients had a fasting insulin/glucose ratio of <0.3. The concentration of TCh did not correlate with any of the metabolic markers in any of the analyzed groups.

**Table 5:** Components of MetS in TS patients during GH treatment.

Component of MetS	1st measurement during GH treatment (n=17)	2nd measurement during GH treatment (n=17)
obesity	0	0
hypertension	3	3
IFG	4	2
low HDL	0	1
hypertriglicerydemia	3	3

MetS, metabolic syndrome; IFG, impaired fasting glucose.

According to the Tanner scale, five patients at first (one - induced maturation with karyotype mos 45,X[85]/46,Xi(X)q10[15], four – spontaneous maturation with types of karyotype - 46,X, i(X)(q10); mos45,X[34]/46,X,r(X)(p22?q)?[16]; and 45,X in two patients) and eight study patients at the second markers determination started puberty (Tanner stage ≥ B2: additionally: one – induced maturation with karyotype 45,X and two spontaneous maturation - mos45,X[7]/46,XX[57] and mos 45,X[7]/46,XX[35]). Heart defects were observed in six patients: coarctation of the aorta (3 patients) and bicuspid aortic valve (3 patients).

## Discussion

We observed that during GH treatment compared to the state before GH treatment, MMP-2 concentration was significantly higher, and we found that the highest values were reproducible in the same patients. It is known that MMP activity is considerably influenced by many secreted growth factors, as it could be an effect of GH (42–44).

Rendeva et al. conducted a study evaluating the concentrations of MMP-2, MMP-9, and VEGF in adults diagnosed with growth deficiency. However, in the case of MMP-2, the study was opposite to ours, as a reduction in the concentration of MMP-2 was observed under the influence of GH (45). Additionally, Rendeva et al. reported that MMP-2 concentration at baseline (before GH therapy) in the study group was higher than the concentration observed in the healthy control group (45), and lower levels of CRP were found in the control group. It is known that inflammation can induce the activation of MMPs (42, 43), as it could be one of the reasons for the high concentration in their study group. According to some reports, the concentration of MMPs increases with age (46), so it is difficult to clearly compare our results with those of Rendeva et al. Unfortunately, we do not have any reports from studies on MMP-2 levels in patients with TS, other than our two previous studies (5) (6), which suggested lower MMP-2 plasma concentration in girls with TS before GH treatment than in healthy girls with short stature.

It is known that MMP-2 plasma concentration can be increased in patients with isolated systolic hypertension (34, 35) and in patients with metabolic syndrome (32, 33). In our study group before GH medication, we had only two patients with hypertension, and none of the patients met the criteria for metabolic syndrome; hence, it is difficult to associate the concentration of this marker with the mentioned parameters.

Regarding the observed possible association of MMP-1 with hypertension among our results, it is worth noting that according to the literature, its expression is increased in human vascular smooth muscle cells exposed to angiotensin II, as has been described in cases of hypertension (47). However, it is not only one connection with metabolic syndrome components, as MMP-1 serum levels are associated with atherosclerotic total plaque burden (46), and its production can be intensified in macrophages during dysglycemia (48) or elevated in plasma in case of hypertriglyceridemia as well as in CRP increase (49). The network of connections identified to date was significant.

In turn, MMP-9 increased production in macprophages and smooth muscle cells was observed during dysglycemia (48) or dyslipidemia (46), and its plasma concentration is elevated in cases of hypertension (50) or obesity (51). It is worth recalling that in our study in the only obese patient before GH treatment, the concentration of MMP-9 was significantly higher than that in the remaining patients (3.5 times higher than the average in the untreated group and almost 2 times higher among GH-treated girls). In the second measurement, we additionally found an IFG in that girl. Perhaps such a high concentration is related to obesity or is an early indicator of carbohydrate disturbances.

All the described connections of MMPs with metabolic disorders suggest that they may serve as biomarkers and potential therapeutic targets for certain vascular disorders.

With respect to VEGF, we found a difference in the concentration of VEGF during the determinations under GH treatment. So far, we have not found any differences in our previous studies: there were no differences between the non-GH patients and healthy short stature girls (6), nor were there any differences in the pilot study where GH patients and healthy short stature girls serving as the control group were enrolled (5). Literature reports on the role of VEGF in TS concern the relationship of its overexpression with fetal hydrops and abnormal endocardial cushion development, and therefore, congenital heart defects. The authors also suggested secondary fibrosis of several organ systems as a consequence of excessive VEGF production (52). As mentioned, the role of VEGF is being studied in the context of carbohydrate-lipid disturbances. Despite the large number of reports on this theme, they are often indispensable or even contradictory. However, it seems that VEGF plays a role in the carbohydrate-lipid area, often intertwined in action from MMPs, enhancing their production (43, 53). As this factor also takes part in many processes, such as angiogenesis, embryogenesis, wound healing, inflammation, tumor metastasis, cardiovascular diseases, or rheumatoid arthritis (21), and its concentration can be influenced by significant familial correlations of its plasma concentration between genetically related individuals (54), it is difficult to confidently point out the reason for the difference between its concentration in girls during GH therapy. However, we want to note there are reports of a decline in VEGF associated with growth hormone treatment (45).

All the markers discussed so far have multiple functions that are intertwined in many areas. They all can have local as well as global effects on the body, are synthesized, among others, in smooth muscle cells or endothelium, and differences in their concentrations can be observed in plasma. It is known that GH, among its other actions, also exerts an effect on the endothelium (55, 56). Taking into account the beneficial effect of GH on endothelial dysfunction, the same effect on cardiovascular disorders (57), its use in TS seems to be particularly beneficial, where research confirms impaired function of the endothelium (56). Therefore, all observed changes during GH therapy should correspond to hypothetically favorable changes in markers concentration.

It remains to discuss the last group of markers analyzed in this study, that is, neurotrophic factors, of which BDNF seems to be more researched. We found no differences in BDNF concentrations during this study. However, in our two previous studies, we observed higher plasma levels of this factor in girls with TS than in healthy girls with short stature (5, 6). This result was also confirmed by Czyzyk et al. (16). Therefore, what leads to higher BDNF concentrations in TS? BDNF seems to be relevant to TS and, according to Farooqui et al., the gene encoding BDNF plays an important regulatory role in TS (58). Moreover, the relationship between BDNF and metabolic disorders cannot be ignored, taking into consideration the assumptions of the hypothesis of metabotropic deficit mentioned in the introduction (12, 13).

In turn, the GDNF concentration was below the limit of detection. The literature suggests that the plasma concentration of GDNF is also included in the metabotropic deficit hypothesis and does not necessarily reflect tissue synthesis (19).

It is difficult to say whether the occurrence of maturation features influenced the values of the determined markers due to the small size of the studied groups. It was not unequivocally found that the highest or the lowest values were found in patients with features of puberty.

We are aware of the limitations of our study as it was performed on a small number of patients. However, it is difficult and time-consuming to gather a sufficient number of patients with a rare syndrome. Despite this limitation, the study remains unique in the context of the analyzed group and selected metabolic markers.

## Conclusion

Understanding the mechanisms of the impact of these markers on the development of metabolic syndrome components is an interesting area of research that seems to be even more important in TS, burdened with more frequent occurrence of metabolic syndrome and cardiovascular diseases. Additionally, the markers discussed here may serve as potential therapeutic targets for metabolic and cardiovascular disorders.

## Data Availability Statement

The raw data supporting the conclusions of this article will be made available by the authors, without undue reservation.

## Ethics Statement

The studies involving human participants were reviewed and approved by The Ethics Committee of the Medical University of Silesia (resolution number KNW/0022/KB1/162/15/16). Written informed consent to participate in this study was provided by the participants’ legal guardian/next of kin.

## Author Contributions

EB and AG designed the study, prepared the database, and wrote the manuscript. JGi monitored the patients and collected the samples for biochemical analysis. JGa analyzed the patient database and wrote the manuscript. MK-F and GH performed the laboratory analyses. TF collaborated in designing the work and performed the laboratory analyses. All authors contributed to the article and approved the submitted version.

## Conflict of Interest

The authors declare that the research was conducted in the absence of any commercial or financial relationships that could be construed as a potential conflict of interest.

## Publisher’s Note

All claims expressed in this article are solely those of the authors and do not necessarily represent those of their affiliated organizations, or those of the publisher, the editors and the reviewers. Any product that may be evaluated in this article, or claim that may be made by its manufacturer, is not guaranteed or endorsed by the publisher.
